# Angular Leaf Spot Resistance *Loci* Associated With Different Plant Growth Stages in Common Bean

**DOI:** 10.3389/fpls.2021.647043

**Published:** 2021-04-13

**Authors:** Caléo Panhoca de Almeida, Jean Fausto de Carvalho Paulino, Gabriel Francesco Janini Bonfante, Juliana Morini Kupper Cardoso Perseguini, Isabella Laporte Santos, João Guilherme Ribeiro Gonçalves, Flávia Rodrigues Alves Patrício, Cristiane Hayumi Taniguti, Gabriel de Siqueira Gesteira, Antônio Augusto Franco Garcia, Qijian Song, Sérgio Augusto Morais Carbonell, Alisson Fernando Chiorato, Luciana Lasry Benchimol-Reis

**Affiliations:** ^1^Centro de Pesquisa em Recursos Genéticos Vegetais, Instituto Agronômico - IAC, Campinas, Brazil; ^2^Centro de Grãos e Fibras, Instituto Agronômico - IAC, Campinas, Brazil; ^3^Laboratório de Fitopatologia, Instituto Biológico - IB, Campinas, Brazil; ^4^Departamento de Genética, Escola Superior de Agricultura “Luiz de Queiroz”, Universidade de São Paulo, Piracicaba, Brazil; ^5^USDA-ARS, Soybean Genomics and Improvement Lab, Beltsville, MD, United States

**Keywords:** *Phaseolus vulgaris* L., *Pseudocercospora griseola*, genome-wide association studies, linkage mapping, disease resistance

## Abstract

Angular leaf spot (ALS) is a disease that causes major yield losses in the common bean crop. Studies based on different isolates and populations have already been carried out to elucidate the genetic mechanisms of resistance to ALS. However, understanding of the interaction of this resistance with the reproductive stages of common bean is lacking. The aim of the present study was to identify ALS resistance *loci* at different plant growth stages (PGS) by association and linkage mapping approaches. An BC_2_F_3_ inter-gene pool cross population (AND 277 × IAC-Milênio – AM population) profiled with 1,091 SNPs from genotyping by sequencing (GBS) was used for linkage mapping, and a carioca diversity panel (CDP) genotyped by 5,398 SNPs from BeadChip assay technology was used for association mapping. Both populations were evaluated for ALS resistance at the V2 and V3 PGSs (controlled conditions) and R8 PGS (field conditions). Different QTL (quantitative trait *loci*) were detected for the three PGSs and both populations, showing a different quantitative profile of the disease at different plant growth stages. For the three PGS, multiple interval mapping (MIM) identified seven significant QTL, and the Genome-wide association study (GWAS) identified fourteen associate SNPs. Several *loci* validated regions of previous studies, and *Phg-1*, *Phg-2, Phg-4*, and *Phg-5*, among the 5 *loci* of greatest effects reported in the literature, were detected in the CDP. The AND 277 cultivar contained both the *Phg-1* and the *Phg-5* QTL, which is reported for the first time in the descendant cultivar CAL143 as ALS10.1^UC^. The novel QTL named ALS11.1^AM^ was located at the beginning of chromosome Pv11. Gene annotation revealed several putative resistance genes involved in the ALS response at the three PGSs, and with the markers and *loci* identified, new specific molecular markers can be developed, representing a powerful tool for common bean crop improvement and for gain in ALS resistance.

## Introduction

Common bean (*Phaseolus vulgaris* L.) is the most important grain that is directly consumed in the human diet, corresponding to half of the grain legumes consumed in the world. The expressive consumption of the grain is mainly due to its nutritional attributes. As it is a legume, common bean associates with nitrogen-fixing bacteria, leading to significantly higher protein concentration than in other species (Broughton et al., 2003). Studies indicate that the species is originally from the American continent (Bitocchi et al., 2012, 2013), and due to the process of evolution and domestication, it has two distinct gene pools known as the Mesoamerican and the Andean (Gepts and Bliss, 1988; Koinange and Gepts, 1992). In some countries in Africa and the Americas, bean provides an average of 15% of total daily calories and 36% of daily protein content (Schmutz et al., 2014). Global production of bean has increased 20.3% since 2012, with a record 30.8 million metric tons in 2017. However, production decreased in 2018 (FAOSTAT, 2021). Brazil is the largest consumer of beans in the world and has the estimated production of the last year around 3.1 million tons (CONAB, 2021), with commercial carioca beans accounting for 70% of the total varieties consumed (Silva et al., 2014). The carioca variety belongs to the Mesoamerican gene pool (Almeida et al., 2020a) and the first cultivar was launched in 1971 by the Instituto Agronômico (IAC, Campinas, SP, Brazil). The new cream-striped cultivar quickly became widely cultivated in Brazil because of its excellent technological qualities and high productivity (Almeida et al., 1971).

The occurrence of diseases is one of the main causes of decrease in agricultural production, and especially so in growing of bean. In certain countries such as Brazil, bean is subject to 45 different diseases caused by multiple pathogens (Borém and Miranda, 2009). Among them, angular leaf spot disease caused by the fungus *Pseudocercospora griseola* (Sacc.) Crous and Braun is found in approximately 80 countries and is considered one of the most devastating and recurrent diseases in the areas of greatest production in Latin America and Africa (Correa-Victoria et al., 1989; Liebenberg and Pretorius, 1997; Stenglein et al., 2003; Sartorato, 2004; Crous et al., 2006; Nay et al., 2019b). ALS can result in losses of up to 70% of production, depending on environmental conditions, the pathogenicity of the isolates, the level of susceptibility of the cultivar, and the stage of plant growth (Schwartz, 1981; Seijas and Sartorato, 1985; Jesus et al., 2001).

Although fungicides are an option for controlling ALS, they are an expensive measure, especially for small producers, who produce most of the bean crop in tropical countries (Nay et al., 2019b). The best effective way to control the disease is through resistant cultivars; the genetic characterization of resistance is of considerable importance for bean breeding (Oblessuc et al., 2012). Several sources of resistance have been reported (Pastor-Corrales and Jara, 1995; Pastor-Corrales et al., 1998; Mahuku et al., 2003; Oliveira et al., 2004; Reis-Prado et al., 2006; Sartorato, 2006; Bruzi et al., 2007; Oblessuc et al., 2012; Keller et al., 2015; Bassi et al., 2017), including the AND 277 cultivar, which is one of the cultivars most used by ALS breeding programs and genetic control studies (Carvalho et al., 1998; Aggarwal et al., 2004; Arruda et al., 2008; Gonçalves-Vidigal et al., 2011; Sanglard et al., 2013; Bassi et al., 2017; Silva et al., 2018).

Due to co-evolution processes between pathogen and host, *P. griseola* can also be divided into Andean and Mesoamerican races, and it is observed that Mesoamerican races infects both Mesoamerican and Andean bean genotypes, while Andean races preferentially infect Andean genotypes (Guzman et al., 1995; Pastor-Corrales and Jara, 1995; Crous et al., 2006). Thus, genetic breeding strategies may use this knowledge to pyramid both Andean and Mesoamerican resistance genes to durable ALS resistance. Futhermore, Andean beans can be used as a source of resistance for introgression of genes to Mesoamerican genotypes, as in the case of the carioca variety (Nay et al., 2019b).

Among the Andean sources of ALS resistance, the AND 277 cultivar contains the *Phg-1* gene (Gonçalves-Vidigal et al., 2011), one of the 5 resistance *loci* associated with ALS and recognized by the Genetics Committee of the Bean Improvement Cooperative (BIC). In addition to *Phg-1*, two other independent and dominant *loci* were reported by the BIC Genetics Committee: the *Phg-2*, mapped on chromosome Pv08 in the México 54 and BAT 332 cultivars (Sartorato, 2002; Namayanja et al., 2006), and *Phg-3*, mapped on Pv04 in the Ouro Negro (Corrêa et al., 2001; Faleiro et al., 2003; Gonçalves-Vidigal et al., 2013). Two other QTL of greater effects were also reported: *Phg-4* of the ALS4.1^UC, AM^ QTL, mapped on chromosome Pv04 in the G5686 and CAL 143 cultivars (Mahuku et al., 2009; Oblessuc et al., 2012; Keller et al., 2015; Souza et al., 2016) and *Phg-5* of the ALS10.1^UC, GS^ QTL, identified on Pv10 in the CAL 143 and G5686 (Oblessuc et al., 2012, 2013; Keller et al., 2015; Souza et al., 2016).

While only 5 *loci* have been reported by the BIC Genetics Committee, several authors have detected quantitative control for the disease, and numerous QTL have already been identified, showing the complex inheritance of ALS resistance (López et al., 2003; Caixeta et al., 2005; Oblessuc et al., 2012; Keller et al., 2015; Perseguini et al., 2016; Bassi et al., 2017; Pereira L. A. et al., 2019, Pereira R. et al., 2019; Librelon et al., 2020). In a recent study, bean accessions evaluated in three PGS and environments regarding degree of resistance to ALS exhibited different resistance levels, as the same cultivar considered resistant in one PGS showed susceptibility in another (Pereira R. et al., 2019).

Genome-wide association study (GWAS) provide several benefits over the QTL mapping approach, including high resolution and use of the genetic diversity available in the accessible germplasm resources for ALS resistance (Perseguini et al., 2016; Fritsche-Neto et al., 2019; Nay et al., 2019b). Many recombinational events and a larger number of SNPs increase the accuracy and narrow the confidence interval of QTL mapping. However, GWAS may generate false positives because of the population structure. Therefore, combining GWAS and linkage mapping would enable utilization of the complementary power of both approaches to identify conservative QTL (Wu et al., 2020).

Several mapping studies on ALS resistance involving different environments, isolates, and mapping populations have already been carried out (Corrêa et al., 2001; Faleiro et al., 2003; Gonçalves-Vidigal et al., 2011, 2013; Oblessuc et al., 2012, 2013; Keller et al., 2015). However, no studies have been performed regarding differences in genetic control of resistance at different PGSs. Thus, the aim of the current study was to identify resistance QTL by linkage and association mapping in three PGSs (V2, V3, and R8) in a greenhouse and field to elucidate the genetic modulation of the degree of resistance throughout the plant growth stages under ALS infection.

## Materials and Methods

### Plant Material

**FIGURE 1:**
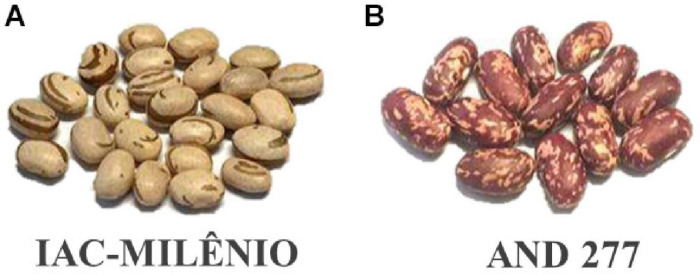
The AM BC_2_F_3_ mapping population parents: **(A)** IAC-Milênio, the Mesoamerican recurrent and susceptible parent; **(B)** AND 277, the Andean donor and resistant parent.

For linkage mapping, a BC_2_F_3_ segregating population ({[(♀AND 277 × ♂IAC-Milênio) × IAC-Milênio] × IAC-Milênio}) was developed at the Common Bean Research Center at the Instituto Agronômico - IAC (Campinas, SP, Brazil). The AM population was composed of 91 inter-gene pool inbred lines selected according to the carioca grain ideotype. To obtain this population, the recurrent parent, IAC-Milênio cultivar, was crossed with the donor parent, AND 277 (Figure 1). The resultant progeny was backcrossed twice with the recurrent parent and selected for the carioca grain type in the BC_2_F_2_ generation.

AND 277 was developed by CIAT (International Center for Tropical Agriculture, Cali, Colombia) through crossing the lines G21720 × BAT1386; it has large seeds and belongs to the Andean gene pool (Aggarwal et al., 2004). It contains resistance genes, such as the **Co*-1^4^* allele, which confers resistance to 21 races of *Colletotrichum lindemuthianum* (Sacc. and Magnus) Briosi and Cavara, the causal agent of anthracnose*;* the *Phg-1 locus*, which confers resistance to *P. griseola* (Gonçalves-Vidigal et al., 2011); and the PWM11^AS^ QTL, associated with resistance to *Erysiphe polygoni* DC, the causal agent of powdery mildew (Bassi et al., 2017). The Mesoamerican IAC-Milênio was released in 2013 by IAC, with an average yield of 2,831 kg / ha^–1^, high grain quality with resistance to seed coat darkening, and resistance to *Fusarium oxysporum* (Carbonell et al., 2014). In addition to high genetic divergence, the AM population has contrasting features for agronomic traits, especially concerning resistance to disease, such as anthracnose (Gonçalves-Vidigal et al., 2011; Carbonell et al., 2014) and ALS (Almeida et al., 2020b).

For GWAS, the CDP validated for association mapping through accurate identification of the *PvTFL1y* gene associated with flowering time, pod maturation, and growth habit by Almeida et al. (2020a) was used. The CDP was composed of 125 carioca Mesoamerican accessions selected from the germplasm bank of the IAC to represent the genetic diversity of the Brazilian carioca bean. The CDP was genotyped by high-throughput genotyping technologies using the Illumina BARCBean6K_3 BeadChip containing 5,398 SNPs (Song et al., 2015), from which 1,979 high-quality SNPs with minimum allele frequency (MAF) greater than 3% were selected and used. Information on the carioca cultivars such as pedigree, year of release, institution of origin, and genotypic data are available in Supplementary Table 1.

### DNA Extraction, Genotyping, and SNP Calling

DNA was extracted from the young leaves of 91 BC_2_F_3_ genotypes and parents using the DNeasy Plant Mini Kit (Qiagen, United States), according to manufacturer’s specifications. The samples were quantified by Qubit fluorometer (Thermo Fisher Scientific, United States) and diluted to 10 μg/μL. A total of 50 ng of each sample was sent to SNPsaurus LLC (Eugene, OR, United States) for generation of the nextRAD (Nextera-tagmented reductively amplified DNA) library and sequencing. The nextRAD method consists of using selective primers to amplify common genomic regions between samples (Russello et al., 2015). First, the DNA is fragmented by the Nextera reagent (Illumina, Inc) containing adapters that are linked to the generated fragments. Then, the fragments are amplified by primers specific to the adapters so that a selective seven-nucleotide chain (TGCAGAG) is added to each amplicon. The resulting fragments are fixed at a selective end and have random lengths, making the amplified DNA of a specific *locus* present in many different sizes. The sequencing of the nextRAD library was carried out in an Illumina HiSeq 4000 sequencer with two 150 bp reading ranges and 20x coverage.

The reads were pre-processed by SNPsaurus for elimination of adapters and alignment with the reference genome *Phaseolus vulgaris v2.1* (Schmutz et al., 2014) through bbduk software (BBMap tools^1^). After pre-processing, genotype data were filtered using the *vcftools* software (Danecek et al., 2011), eliminating monomorphic *loci*, missing data higher than 20%, heterozygous and multiallelic *loci*, and depth below 4x. The heterozygous *loci* were eliminated as the expected heterozygosity in the mapping population is negligible, thus enabling phenotypic evaluation of the AM population in future generations, without the need to change the expected frequency of recombination for the association. High-quality filtered SNPs were used to construct the AM linkage map. The raw sequencing reads have been deposited in the NCBI Sequence Read Archive^2^ under the Project Number PRJNA705743.

For construction of the genetic map, a set of 23 molecular markers, previously characterized as polymorphic for the parents and associated with resistance *loci* in previous studies, were also used (Table 1). The PCR products (i.e., amplicons) from SCAR (Sequence Characterized Amplified Region) markers (Paran and Michelmore, 1993) and STS (Sequence-Tagged Site) (Olson et al., 1989) were separated on 1.2% agarose gels, and Single Sequence Repeats (SSRs - Tautz, 1989) were genotyped by capillary electrophoresis using an automated 96 capillary Fragment Analyzer^TM^ system (Agilent Technologies, Advanced Analytical Technologies, AATI, Santa Clara, CA, United States) with the DNF-905 double-stranded DNA reagent kit.

**TABLE 1:** Molecular markers previously associated with angular leaf spot resistance QTL used for the genotyping of the AM population (91 BC_2_F_3_).

Marker	Type^1^	LG^2^	QTL	References
CV542014	STS	1	*Phg-1*	Gonçalves-Vidigal et al. (2011)
TGA1.1	STS	1	*Phg-1*	Gonçalves-Vidigal et al. (2011)
IAC134	SSR	2	ALS2.1^uc^	Oblessuc et al. (2012)
IAC18b	SSR	2	ALS2.1^uc^	Oblessuc et al. (2012)
BM159	SSR	3	ALS3.1^UC^	Oblessuc et al. (2012)
FJUNA19	SSR	3	ALS3.1^UC^	Oblessuc et al. (2012)
PVBR21	SSR	3	ALS3.1^UC^	Oblessuc et al. (2012)
BMd9	SSR	4	ALS4.1^GS/UC^	Oblessuc et al. (2012)
Pv-ctt001	SSR	4	–	Mahuku et al. (2009)
BM175	SSR	5	ALS5.2^UC^	Oblessuc et al. (2012)
BMd53	SSR	5	ALS5.1^UC^	Oblessuc et al. (2012)
Pvat006	SSR	5	ALS5.2^UC^	Oblessuc et al. (2012)
PVBR124	SSR	5	ALS5.1^UC^	Oblessuc et al. (2012)
IAC261	SSR	5	ALS5.2^UC^	Oblessuc et al. (2012)
ATA244	SSR	10	ALS10.1^DG/UC^	Oblessuc et al. (2013)
BM157	SSR	10	ALS10.1^DG/UC^	Oblessuc et al. (2013)
GATS11b	SSR	10	ALS10.1^DG/UC^	Oblessuc et al. (2013)
PVM127	SSR	10	ALS10.1^DG/UC^	Oblessuc et al. (2013)
PVM22	SSR	10	ALS10.1^DG/UC^	Oblessuc et al. (2013)
IAC54	SSR	10	ALS10.1^DG/UC^	Oblessuc et al. (2013)
IAC61	SSR	10	ALS10.1^DG/UC^	Oblessuc et al. (2013)
SBA16	SCAR	–	–	Queiroz et al. (2004)
SH13	SCAR	–	–	Queiroz et al. (2004)

*^1^molecular marker type.*

*^2^linkage group.*

### Evaluation of ALS Resistance at Different Plant Growth Stages

The AM population and the CDP were evaluated under controlled inoculation conditions at two PGSs. The first experiment was at the V2 stage (i.e., primary expanded leaves) and the second at the V3 stage (i.e., first expanded three leaf stage). The design adopted was completely randomized blocks with three replications. Each block was composed of two plots, and the plot consisting of 1 plastic pot (11 × 8 × 9 cm) filled with plant substrate (Biomix^®^) with two plants, for a total of four plants per block. The AM population evaluations in the V2 and V3 stages occurred in the BC_2_F_3_ generation. In both experiments, the parents were included among the treatments as check cultivars.

Monosporic isolates kindly provided by the Department of Biology at the Federal University of Lavras (UFLA, MG, Brazil) were used for inoculation, and their physiological races were characterized through the differentiating series proposed by Pastor-Corrales and Jara (1995). The plants remained in the greenhouse until they reached the determined PGS and, after that, they were transported to an inoculation chamber, where they were kept under controlled temperature (23°C ± 2°C), relative humidity (95% to 100%), and photoperiod (12 h) until the final evaluation.

Inoculation at the V2 stage occurred nine days after planting, following the methodology proposed by Pereira et al. (2011), with an inoculum concentration of 4 × 10^–4^ conidia mL^–1^, while inoculation at the V3 stage occurred 15 days after planting, following the methodology described by Pastor-Corrales et al. (1998). Severity assessment occurred 15 days after inoculation (DAI) using the diagrammatic scale proposed by Librelon et al. (2015) for the V2 stage, and 18 DAI using the CIAT diagrammatic scale described by Van-Schoonhoven and Pastor-Corrales (1987) for the V3 stage. Both scales range from 1 to 9, where 1 represents the absence of symptoms and 9 the maximum degree of infection, discriminating the plants into resistant (score ≤ 3), moderately resistant (scores 4 to 6), and susceptible (≥ 7).

A third experiment to assess ALS resistance at a late stage of plant reproductive growth was performed in the field using the AM population and the CDP. The experiments were carried out at the Fazenda Santa Eliza experimental station at IAC, during the winter season, with the AM population being sown in April / 2018 and the CDP in March / 2020. The experimental plot was composed of 1-m rows with 10 plants, and the between-row spacing was 0.5 m. A completely randomized block experimental design was once more adopted, with four replications, and AND 277 and IAC-Milênio were included as check cultivars. To ensure homogeneity of the infection, artificial inoculation with the *P. griseola* isolate, at a concentration of 2 × 10^4^ conidia mL^–1^, was performed 30 days after sowing. Phenotypic evaluation was carried out at the R8 stage, at approximately 35 DAI, using the diagrammatic scale proposed by Van-Schoonhoven and Pastor-Corrales (1987). The six central plants of each plot were evaluated, and the final score of each genotype was given by the arithmetic average of the plants evaluated.

### Statistical Analyses of the Phenotypic Data

The descriptive statistics and variance analysis (ANOVA), as well as genetic parameters, were estimated using the RBio software (Bhering, 2017). The normality of the residues of ANOVA was tested by the Shapiro-Wilk test (Shapiro and Wilk, 1965) and, in the absence of normality, the data were normalized by the Box-Cox transformation (Box and Cox, 1964). The effects of the different sources of variation were considered significant by the *F*-test when *p* < 0.05.

### Linkage Map Construction and QTL Association

The genetic map was constructed using the OneMap package (Margarido et al., 2007), modified according to the genetic structure of the AM population. The markers were evaluated for redundancy and tested for the expected Mendelian segregation for the population, using the chi-square test and genotype frequencies of 1/8:7/8. After elimination of redundant markers and with segregation distortion, the recombination fractions were calculated using the exhaustive search algorithm “grid search,” based on maximizing the multinomial likelihood function with the expected frequencies of the genotypes for the BC_2_F_3_ population.

Linkage groups were obtained by combining information on recombination frequencies and information from alignment with the *Phaseolus vulgaris* v2.1 (Schmutz et al., 2014) reference genome. Once the linkage groups were formed, the markers were ordered using combinations of the *COMPARE*, *TRY*, and *MDS* algorithms (Preedy and Hackett, 2016) in rounds, and by manual insertions and deletions based on the visual pattern of the recombination fraction and LOD score heatmaps. The orderings were defined by the combination of the smallest chromosome size, maximum likelihood value, and heatmaps with the best visual patterns. Then, the recombination fractions were re-estimated using the multipoint approach (Wu et al., 2002) by Markov models (Baum et al., 1970). The initiation probabilities were modified according to the expected allele frequencies in the population, and the transition probability matrix was modified following the generalization proposed by Jiang and Zeng (1997).

Markers without chromosome information (i.e., scaffolds) were inserted in all possible positions for all chromosomes, using the *TRY* algorithm. For each insertion, the multipoint estimates were recalculated and the variation in chromosome size and the variation in likelihood promoted by the insertion of the marker were evaluated. The most likely chromosome for each marker was obtained by the combined evaluation of both values. After defining the most likely chromosomes for each marker, they were reinserted in their most likely position according to the *TRY* algorithm. The same strategy was used for the SSR, STS, and SCAR markers, however, considering the “*a priori*” information of the chromosomes to which they were mapped in previous studies (Table 1). For markers that do not have chromosomal information, the same strategy used for the scaffolds was adopted.

After construction of the genetic map, the probabilities of the genotypes of the putative QTL were obtained by the hidden Markov chain, with steps every 1 cM. The adjusted values of the traits were progressively used to fit the QTL mapping models. Initially, markers with significant effects were identified using a fixed linear regression model. Then, the markers detected as significant were used as cofactors in the composite interval mapping (CIM) model, proposed by Zeng (1994), considering a sliding window of 20 cM. The putative QTL were tested for statistical significance by comparing their LOD values with a threshold obtained by 1,000 permutations (Churchill and Doerge, 1994), considering the significance level of 5%. The QTLs identified in the CIM were used as a starting point for the multiple interval mapping (MIM) procedure, based on the model proposed by Kao et al. (1999).

The strategy for obtaining the most appropriate QTL model involved three steps: searching for QTL, testing effects, and selecting the model. Starting from the model with the QTL identified by CIM, new QTL were searched for along the genome, also using a 20 cM sliding window. The QTL with the highest LOD values were inserted into the model, and then all QTL were tested for conditional significance. Both models (complete and reduced) were also compared regarding the Akaike information criterion - AIC (Akaike, 1974). If a QTL had a non-significant effect or an AIC value higher than the reduced model, it was removed from the model. The procedure was repeated until no QTL was added or removed from the model. Thus, the final model was selected, and the positions and effects of the QTL were re-estimated, with the variation explained by each QTL (*R*^2^). The genetic map was plotted using the *draw*_*map2* function from the OneMap package (Margarido et al., 2007).

### Genome-Wide Association

For association mapping, a fixed and random model of the Circulating Probability Unification - FarmCPU model (Liu et al., 2016) was used. The package explores the MLMM model (Multi-*Locus* Mixed-Model) and performs the analysis in two interactive steps: A Fixed Effect Model (FEM) is applied first, followed by a Random Effect Model (REM). Both models were repeated interactively until no significant SNP was detected. As demonstrated by Almeida et al. (2020a), a genetic structuring matrix was not used, due to the absence of a sub-cluster in the panel. The phenotypic matrix was given by the genotypic values estimated in each index by a Restricted Maximum Likelihood / Best Linear Unbiased Estimator (REML / BLUE) using the Be-Breeder package (Matias et al., 2018).

The *p*-value threshold of each SNP was determined by the resampling method using the function *FarmCPU.P.Threshold*. Each trait was permuted 1,000 times to break the relationship with the genotypes, and then the random association among all SNPs to the phenotype was estimated. The minimum *p*-value obtained among all SNPs for the 1,000 repetitions was recorded and then the 95% quantile from all minimum *p*-values was defined as the *p*-value threshold (Churchill and Doerge, 1994). Bonferroni test (Bonferroni, 1963) was also used as a threshold for the output in the Manhattan plot (cutoff α = 0.05).

### Identification of Candidate Genes

The CDP had an average distance for the standard linkage disequilibrium (LD) decay threshold (*r*^2^ = 0.2) at 0.59 Mb, considering all chromosomes (Almeida et al., 2020a). However, for effective determination of the size interval of each quantitative trait nucleotide (QTN) for accurate identification of candidate genes, a 10 Mb flanking region of the significant SNPs was tested for LD through the Gaston package (Dandine-Roulland and Perdry, 2017), with the interval size given by the size of the haplotype blocks in LD. The SNP genomic positions and the candidate genes were inferred using the *Jbrowse* Phytozome v11.0 (Goodstein et al., 2012) and the *Phaseolus vulgaris* v2.1 genome (Schmutz et al., 2014). The final map contained all identified *loci* and their respective molecular markers, as well as the location of the 376 putative resistance-associated genes encoding nucleotide-binding and leucine-rich repeat domains collectively known as NB-LRR (NL), identified by Schmutz et al. (2014). These data were plotted using the *MapChart* 2.32 software (Voorrips, 2002).

## Results

### AM Linkage Map

The sequencing of the nextRAD library generated an average of 4,611,760 high quality reads per genotype, totaling 428,893,724 reads. Considering the reads that aligned with the reference genome, only 7 lines had an alignment rate below 80%, probably due to contamination of the plant tissue used for DNA extraction. The BLAST of these sequences with the NCBI database revealed homology with bacterial species, such as *Pantoea agglomerans*, which is commonly found and isolated from plant surfaces, with greater abundance in leaves and seeds (Iimura and Hosono, 1996).

After eliminating the reads not aligned with the reference genome, a total of 791,361 SNPs were identified, of which 1,091 high-quality, homozygous, non-redundant SNPs with no deviation from the expected Mendelian segregation (1/8:7/8) for the AM population were used to construct the genetic linkage map. In addition to the 1,091 SNPs, an additional 23 molecular markers were used for linkage analysis (Table 1), resulting in a genetic map with a total length of 1,923.16 cM (Supplementary Figure 1). The largest chromosome had a length of 355.33 cM (Pv11) and the smallest, 35.93 cM (Pv06). The 1,114 markers were well distributed over the 11 chromosomes of the species, with an average of 101 markers per chromosome (Table 2). The average distance between markers for the 11 chromosomes was 1.90 cM; the Pv06 chromosome had the highest saturation (i.e., mean distance of 0.58 cM) and Pv01 had the lowest (i.e., mean distance of 5.49 cM). The genetic map with the genotypic data of the AM population is available in Supplementary Table 2.

**TABLE 2:** Molecular marker distribution, length of each linkage group, and the average distance between the 1,114 markers used to estimate the genetic linkage map for the AM population (AND 277 × IAC-Milênio) through the OneMap package.

Chromosome	SNPs	Length (cM)	Mean distance^1^ (cM)
Pv01	48	263.76	5.49
Pv02	52	180.27	3.47
Pv03	164	179.95	1.1
Pv04	180	309.4	1.72
Pv05	122	262.2	2.15
Pv06	61	35.93	0.59
Pv07	74	55.5	0.75
Pv08	89	129.21	1.45
Pv09	58	75.17	1.3
Pv10	74	76.43	1.03
Pv11	192	355.33	1.85
Total	1,114	1,923.16	1.90

*^1^mean distance between markers.*

### Phenotypic Resistance to ALS

**FIGURE 2:**
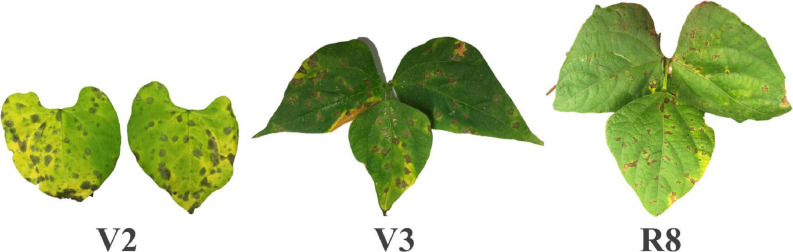
Necrotic spots in angular shape and chlorosis, characteristic symptoms of angular leaf spot disease in the resistance evaluations for the V2, V3, and R8 plant growth stages.

Characterization of the physiological race of the isolate, following the methodology proposed by Pastor-Corrales and Jara (1995), indicated race 31-31, since the Andean differentiating cultivars Don Timoteo [binary value (bv) = 1], G11796 (bv = 2), Bolon Bayo (bv = 4), Montcalm (bv = 8), and Amendoim (bv = 16) and the Mesoamerican differentiating cultivars Pan 72 (bv = 1), G 2858 (bv = 2), Flor de Mayo (bv = 4), Mexico 54 (bv = 8), and BAT 332 (bv = 16) were susceptible. Angular necrosis patches, a characteristic symptom, confirmed occurrence of the disease in all experiments (Figure 2). As expected, the parents of the AM population showed contrasting features for ALS resistance in all the evaluations, with an average score for AND 277 = 1.6 ± 0.3 and for IAC-Milênio = 6.2 ± 0.7 (Table 3).

**TABLE 3:** Estimates of means, standard deviations, percentage of resistance, normality test, experimental accuracy, and broad-sense heritability for angular leaf spot resistance evaluations in the V2, V3, and R8 stages using the AM population and the carioca diversity panel.

Sources of Variation	AM POPULATION			CDP		
	V2	V3	R8	V2	V3	R8
Genotypes (μ)^1^	6.05*** ± 1.8	5.15*** ± 1.4	4.89*** ± 1.6	3.98*** ± 1.4	2.46** ± 1.3	3.25*** ± 1.5
Variation	1–9	1–9	2–9	1–7	1–7	1–7
IAC-Milênio (μ)	7.5 ± 0.7	5.3 ± 1.4	6 ± 0.8	6.5 ± 0.6	6.3 ± 0.8	5.8 ± 0.9
AND 277 (μ)	1.7 ± 0.3	1.6 ± 0.1	1.5 ± 0.5	1.2 ± 0.2	1.1 ± 0.1	1.4 ± 0.3
Resistant (%)^2^	3.2	26.3	7.6	25.6	75.2	52.1
Shapiro -Wilk^3^	0.25^ns^	0.89^ns^	0.11^ns^	0.06^ns^	0.09^ns^	0.18^ns^
Accuracy	0.75	0.78	0.92	0.95	0.94	0.83
h^2^	0.56	0.65	0.85	0.87	0.88	0.65

^1^estimates of means and the ANOVA significance result, where ***, **, ns: significant at 0.001, 0.01, and non-significant by the F test.

*^2^percentage of plants resistant to ALS (score ≤ 3).*

*^3^ ns = non-significant for the Shapiro-Wilk test and the data have normal distribution.*

The V2 stage experiment showed the highest degree of disease severity for both groups evaluated, the AM population and CDP. The wide variability of the AM population and the CDP was confirmed by the high significance of the ANOVA test for all evaluations (*p* < 0.001), validating the use of both sets for analysis of ALS resistance mapping. Broad sense heritability (*h*^2^) ranged from 0.56 to 0.88; the lowest estimation was for the AM population at the V2 stage (*h*^2^ = 0.56) and the highest was for the CDP at the V3 stage (*h*^2^ = 0.88). The number of resistant plants (score < 3) varied among the three PGSs, with the V3 stage showing the largest number of resistant plants (AM = 26.3% and CDP = 75.2%) and the V2 stage the smallest number (AM population = 3.2% and CDP = 25.6%). Phenotypic data for all experiments are shown in Supplementary Table 1, evaluation of the CDP population, and Supplementary Table 2, evaluation of the AM population.

### Genetic Loci Identified by Linkage and Association Mapping

Through MIM, eight QTL showed high significance (*p*-value < 0.003) for the three PGSs (Table 4), which were named according to Pedrosa-Harand et al. (2008). The ALS10.2^AM^ QTL, mapped in Pv10, flanked by Marker851 and Marker852 (range: 2.91 to 15.63 cM; estimated position: 4.05 cM) at the V2 stage, had the highest LOD value and explained 34% of the phenotypic variance (*R*^2^). For evaluation in the V3 stage, four QTL on different chromosomes were identified. The first on chromosome Pv01, ALS1.1^AM^ (range: 10.08 to 19.32 cM; estimated position: 18.48 cM), explained ∼12.5% of the phenotypic variance, followed by ALS5.1^AM^ (range: 151.8 to 154.24 cM; estimated position: 153.43 cM) and ALS10.1^AM^ (range: 38.79 to 42.27 cM; estimated position: 39.95 cM), both explaining approximately ∼10%, and finally ALS9.1^AM^ (range: 9.3 to 17.82 cM; estimated position: 8.99 cM) on Pv09 explaining only ∼4.2% of the resistance. Considering the R8 stage, the ALS10.1^AM^ QTL that was significant at the V3 stage was also significant at R8, explaining approximately ∼5.3% of the phenotypic variance. Two other QTLs with *R*^2^-values estimated at ∼9% were identified: the first ALS3.1^AM^ (range: 151.31 to 156.29 cM; estimated position: 154.42 cM) on Pv03 and the second ALS11.1^AM^ (range: 5.72 to 25.32 cM; estimated position: 8.99 cM) on Pv11. Although the ALS10.1^AM^ QTL identified at the V3 and R8 stage had different estimated positions, both were flanked by the same markers (Marker910 and Marker911) in mapping, showing that it is a single lengthy QTL.

**TABLE 4:** Angular leaf spot resistance QTL mapped by multiple intervals (MIM) at different growth plant stages, using the AM population (AND 277 × IAC-Milênio).

PGS^1^	QTL^2^	Pv^3^	EPI (cM)^4^	Flanking Marker	LOD	*R*^2^ (%)	AE^5^	SE^6^	*p*-value	No. RG^7^
V2	ALS10.2^DG, UC, GS, AS, AM^	10	4.05 (2.91-15.63)	Marker851 Marker852	8.04	34.04	1.53	0.23	0.0000	0
V3	ALS1.1*^*AR*^*^, CY, AM^	1	18.48 (10.08-19.32)	Marker2 Marker3	2.59	12.46	0.98	0.28	0.0008	4
V3	ALS5.2^UC, GS, AS, AM^	5	153.43 (151.8-154.24)	Marker524 Marker525	2.98	10.27	0.55	0.15	0.0003	17
V3	ALS10.1^DG, UC, GS, AS, AM^	10	39.95 (38.79-42.27)	Marker910 Marker911	2.03	10.42	1.28	0.42	0.0030	13
V3	ALS9.1^GS, AM^	9	11.62 (9.3-17.82)	Marker799 Marker800	1.95	4.24	0.61	0.21	0.0037	3
R8	ALS11.1^AS, AM^	11	8.99 (5.72-25.32)	Marker933 Marker934	2.47	9.53	0.77	0.23	0.0010	7
R8	ALS3.1^UC, AM^	3	154.42 (151.31-156.29)	Marker245 Marker246	2.37	9.12	0.59	0.18	0.0012	10
R8	ALS10.1^DG, UC, GS, AS, AM^	10	35.9 (33.58-37.64)	Marker910 Marker911	2.15	5.28	0.86	0.27	0.0021	13

*^1^plant growth stage.*

*^2^QTL were named according to Pedrosa-Harand et al. (2008).*

*^3^chromosomes.*

*^4^estimated position and interval QTL.*

*^5^additive effect.*

*^6^standard error.*

*^7^number of resistance genes in the physical interval.*

**FIGURE 3:**
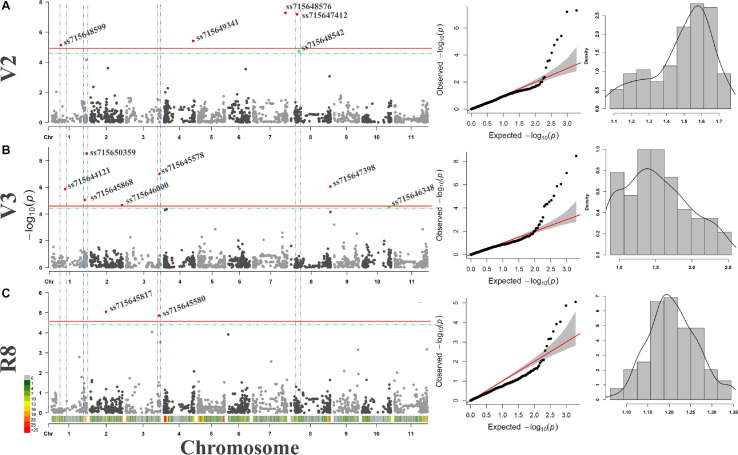
**(A)** Manhattan-plots, QQ-plots, and histograms for GWAS analyses using the carioca diversity panel genotyped by 1,979 SNPs and evaluated for resistance to angular leaf spot disease in the V2 **(A)**, V3 **(B)**, and R8 **(C)** plant growth stages. Regions with more than one significant SNPs for one or more stages are delimited by the blue dashed line. The threshold defined by the Bonferroni (cutoff α = 0.05) test and by the 1,000 permutations are plotted in red and green, respectively.

Similar to linkage mapping, different QTNs associated with resistance to ALS for the three PGSs and different environmental conditions were identified by GWAS using the CDP (Figure 3). In general, 14 SNPs were significant according to the threshold defined by 1,000 permutations (Table 5), of which two had a lower *p* value than the Bonferroni test. The V2 stage revealed one significant SNP on chromosome Pv01, Pv04, and Pv07, and two on Pv08. In the V3 stage, three SNPs were identified on Pv01 and one on Pv02, Pv03, Pv08, and Pv10. Finally, two SNPs were significant for the R8 stage, one on Pv02 and another on Pv03.

**TABLE 5:** GWAS results: Significant SNPs for association mapping using the carioca diversity panel evaluated phenotypically for resistance to angular leaf spot in the V2, V3, and R8 plant growth stages. The effective size of each QTN (start and end) was determined by the interval size of the haplotype blocks containing the markers in LD.

PGS^1^	QTN name	SNP	Pv^2^	Position	QTN start	QTN end	*p-*value	MAF^3^	Effect	No. RG^4^
V2	QTN1.1	ss715648599	1	13596763	–	–	7E-06	0.06	0.11	0
	QTN4.1	ss715649341	4	43649790	43.3	43.8	4E-06	0.14	0.07	3
	QTN7.1	ss715648576	7	48549690	48.3	–	5E-08	0.43	0.07	0
	QTN8.1	ss715647412	8	9296154	6.6	13.2	6E-08	0.23	−0.11	26
	QTN8.2	ss715648542		12274560			2E-05	0.18	−0.09	
V3	QTN1.2	ss715644121	1	20172472	15.8	20.5	1E-06	0.05	−0.28	3
	QTN1.3	ss715645868	1	49487812	–	49.7	9E-06	0.21	0.16	1
	QTN1.4	ss715650359	1	52176970	–	–	3E-09	0.12	0.27	0
	QTN2.2	ss715646000	2	48072185	46.6	–	2E-05	0.45	−0.12	7
	QTN3.2	ss715645578	3	50018570	49.9	50.1	1E-07	0.19	0.17	1
	QTN8.3	ss715647398	8	59270646	–	59.4	9E-07	0.28	−0.17	11
	QTN10.1	ss715646348	10	40164727	40.0	40.3	3E-05	0.08	−0.21	1
R8	QTN2.1	ss715645817	2	24571804	24.0	29.3	9E-06	0.28	−0.02	36
	QTN3.1	ss715645580	3	50004446	49.9	50.1	1E-05	0.15	0.02	1

*^1^plant growth stage.*

*^2^chromosomes.*

*^3^minimum allele frequency.*

*^4^number of resistance genes in the physical interval.*

### Candidate Genes for ALS Resistance

**FIGURE 4:**
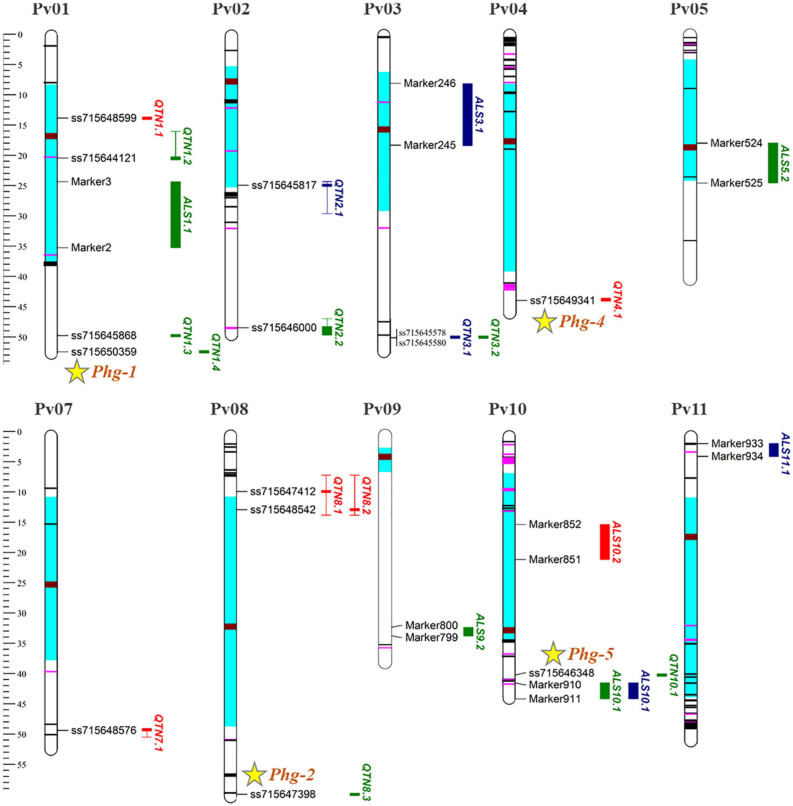
Genetic mapping with the ALS resistance *loci* in different plant growth stages. The significant QTL and QTN represented by red, green, and blue bars on the right side of the chromosomes for the V2, V3, and R8 stages, respectively. The effective interval size of the QTN is given by the size of the haplotype blocks in LD and is represented by the length of the bar, with the filled part being the physical position of the significant SNP. The position of NB-LRR (NL) resistance genes containing nucleotide-binding and leucine-rich repeat domains are marked in black (CNL sequences) and pink (TNL sequences) according to Schmutz et al. (2014). Centromeric and pericentromeric regions are highlighted in brown and light blue. Physical positions provided in megabase pairs (Mb), according to reference genome.

All ALS resistance *loci* identified for the three PGSs and different environmental conditions, using both linkage and association mapping with their respective SNPs, were plotted graphically (Figure 4), with the position of the NB-LRR (NL) resistance genes and the approximate location of the five *loci* named from *Phg-1* to *Phg-*5 and recognized by the BIC Genetics Committee, according to Nay et al. (2019a). In general, the *loci* located close to the centromeric chromosome region had an effective interval larger than those positioned in the distal regions. The QTL intervals were larger than the QTN, such as the ALS1.1^AM^ QTL with 10.9 Mb and the QTN1.2 with 4.7 Mb, both mapped for the V3 stage of evaluation in the pericentromeric region of the Pv01 chromosome.

In addition to the NB-LRR (NL) genes, other genes involved in disease resistance mechanisms were positioned within the *locus* intervals known as “Protein kinase superfamily” (e.g., Phvul.008G083000), “Leucine-rich repeat (LLR) protein family” (e.g., Phvul.002G307200), “Disease resistance - responsive protein family (e.g., Phvul.002G115700)” and “cysteine-rich receptor-like protein kinase - RLK (e.g., Phvul.008G077500)”. The highest number of disease resistance genes was identified for the QTN2.1 *locus*, with an interval of 5.3 Mb and 36 resistance genes (Table 5). The QTN8.1 and QTN8.2 *loci* were 3 Mb distant from each other. However, LD analysis showed that both were in LD for an interval of 6.6 Mb, with the largest *locus* identified by GWAS. In all, 26 resistance genes were identified for this wide *locus*, it being the only one with genes that encode “cysteine-rich RLK” (4). All genes identified in the current study are shown in Supplementary Table 3, highlighting the genes related to disease resistance with genetic annotation.

## Discussion

### Genetic Map Derived From a Contrasting Biparental Cross

The quality of genotyping by sequencing (GBS), the availability of a reference genome for the species, the genetic distance between the parents, and the implementation of the expected recombination frequencies of the AM population in the OneMap package (Margarido et al., 2007) are factors that contributed to the quality of the AM linkage map, making up for reduced population size. In addition, this is the first study reported on plants using the nextRAD approach (*SNPsaurus* LCC), which differs from other GBS by its reliance on selective PCR to reduce genomic complexity instead of restriction enzymes. It showed considerable potential for genetic mapping studies in common beans (Russello et al., 2015; Kia et al., 2017). However, the low number of SNPs employed in the construction of the AM map (1.017) compared to the total number of SNPs identified is mainly due to the stringency of the filters applied to the *SNPcalling* and the elimination of redundant markers.

Although the total length of the map was greater than expected for the bean genome, approximately 1,200 cM (Vallejos et al., 1992), several genetic maps have already been reported that were even larger (Blair et al., 2003; Galeano et al., 2009; de Campos et al., 2011; Bassi et al., 2017). Galeano et al. (2011) proposed a consensus map for bean estimated at 2,041 cM using 1,010 molecular markers, which was used to identify QTLs associated with the most diverse agronomic traits (Diaz et al., 2017). Oblessuc et al. (2012) and Bassi et al. (2017) also used a genetic map with longer sequences to identify QTLs associated with resistance to ALS, sequences estimated at 1,865.9 cM and 1,515.2 cM, respectively. More recently, Silva et al. (2018) estimated a saturated map using 1,962 SNPs genotyped in recombinant inbred lines (AND 277 × Rúda-carioca, AR population). The AR genetic map was estimated at 1,081.98 cM, with an average of 178 markers per chromosome and 0.87 cM average distance.

### Degrees of Resistance Throughout the Plant Growth

In the case of evaluation of ALS resistance using genotypes derived from different gene pools, the use of a *P. griseola* isolate classified as Mesoamerican is necessary, since it has the ability to infect both Andean and Mesoamerican germplasm sources (Guzman et al., 1995; Pastor-Corrales and Jara, 1995). AND 277 showed resistance in all evaluations (scores < 3), confirming the high degree of resistance reported in previous studies (Carvalho et al., 1998; Gonçalves-Vidigal et al., 2011; Bassi et al., 2017; Almeida et al., 2020b). The transgressive segregation observed in ALS resistance for the AM population was also reported by Oblessuc et al. (2012) and Bassi et al. (2017) for others segregating populations. According to Beebe et al. (2008), transgressive segregation occurs when genotypes that have complementary genes with additive effects are hybridized, resulting in greater or lesser expression of the phenotype.

Regarding estimated heritability, similar values were reported by Oblessuc et al. (2012), who estimated *h*^2^ ranging from 0.51 to 0.81 for ALS resistance evaluated in different environments. Bassi et al. (2017) reported similar heritability for the AS population in assessing resistance to ALS at the V3 stage (*h*^2^ = 0.62), and Librelon et al. (2020) as well for the recurrent selection progenies evaluated in the V2 stage (*h*^2^ = 0.64). A smaller number of genotypes were considered resistant in evaluation in the V2 stage, and it may be considered a phenological stage for moderate selection. With rejection of the most susceptible genotypes in the V2 stage, more careful evaluation can be carried out in the V3 evaluation and, if possible, in field evaluation. Differential expression of genes in different environments may be explained by the occurrence of epigenetic events (Pereira R. et al., 2019). However, Pereira R. et al. (2019) reported that 30.5% of the cultivars showed resistance to ALS in the V2 stage, whereas only 6.9% were resistant in the V3 stage.

The difference in degrees of resistance throughout the different PGSs of both sets manifested the complexity of resistance to *P. griseola*, confirming the idea that, in earlier stages, genotypes tend to be more susceptible. Da Costa et al. (2006), when assessing the resistance of soybean cultivars at different PGSs to a fungus that also affects plant shoots (*Colletotrichum truncatum*), reported that the plants were more susceptible in early stages, since the leaf area affected is greater. Garcia and Juliatti (2012) also reported that the average severity of *Sclerotinia sclerotiorum* in commercial soybean cultivars decreased as the PGSs evaluated advanced. For that reason, when proposing the diagrammatic scale for evaluation of ALS resistance in the V2 stage of bean, Librelon et al. (2015) reported that the plants tend to have more severe symptoms in early plant growth stages. Da Costa et al. (2006) explained this phenomenon as a “resistance of the adult plant” mechanism and, according to Gieco et al. (2004), it is essential to evaluate disease resistance in more than one PGS to make correct classification.

### QTL Associated With ALS Resistance

In bean, ALS resistance is reportedly controlled by five major resistance *loci*, named *Phg-1* to *Phg-5* (Souza et al., 2016; Nay et al., 2019a). However, several studies have shown the complexity of genetic resistance to ALS, and many *loci* associated with resistance have been reported (Oblessuc et al., 2012, 2013, 2015; Keller et al., 2015; Perseguini et al., 2016; Bassi et al., 2017). Recently, Nay et al. (2019b) showed that for *Phg-2*, Andean haplotype groups have greater susceptibility to ALS than Mesoamerican groups, confirming the difference in resistance in the gene pools.

The large number of physiological races of *P. griseola* and its high variability in genetic composition and virulence have been reported by several studies (Guzman et al., 1995; Pastor-Corrales et al., 1998; Aggarwal et al., 2004; Sartorato, 2004; Ddamulira et al., 2014; Rezene et al., 2018). In addition, Pereira et al. (2015) showed the existence of great variability in virulence for isolates classified as the same race and studies have shown that genetic control of ALS resistance differs depending on the plant tissue affected (Borel et al., 2011; Rezende et al., 2014). Other studies also reported the genotype × environment interaction for evaluations under conditions of natural occurrence of the pathogen, showing a large environmental effect on control of resistance, corroborating the quantitative profile of the disease (Amaro et al., 2007; Oblessuc et al., 2012; Pereira R. et al., 2019). Pereira R. et al. (2019) evaluated 144 bean accessions for ALS resistance in three different PGSs, and showed that the percentage of genotypes considered resistant to ALS ranging from 7% to 31%, and that the variation in the percentage of coincidence as low for qualitative inheritance.

Regarding genetic mapping studies, several authors have shown quantitative control of the disease, both for assessments in a controlled environment and under natural conditions of infection (Oblessuc et al., 2012; Keller et al., 2015; Perseguini et al., 2016; Bassi et al., 2017). Given that many disease resistance genes in bean exist in gene clusters at complex *loci* (Kelly et al., 2003), it is becoming increasingly important to understand the physical arrangement and sequence diversity of disease resistance gene families in the crop (Miklas et al., 2006).

In the current study, considering QTL analysis, seven different *loci* associated with ALS resistance for the three PGSs were mapped on six different chromosomes. One noteworthy point is that the reduced sample size of the AM population might have reduced the statistical power for QTL detection and mapping resolution. Another point is the fact that the AM population was selected for the ideotype of carioca commercial grain, which may have resulted in elimination of genotypes with resistance *loci* since the carioca type seed coat is also a trait related to the susceptible parent. Due to the small number of the population (*n* = 91), the statistical power to detect a QTL with lesser effect is less than 3%, according to Xu (2003), and the estimated effect of a QTL is inflated 10-fold due to the Beavis effect (Beavis, 1998). Another limiting factor for the number of QTL identified is the insufficiency of genetic diversity in biparental populations, causing many genetic *loci* to be lost (Liu et al., 2020). We tried to overcome this problem by validating the AM detected *loci* on a diversity panel (CDP). In this sense, using two different mapping approaches to enable greater statistical power in the identification of significant *loci* and validation of the results was the best way out and proved to be effective.

The QTL with the greatest effect (*R*^2^ = 34%) was ALS10.2^AM^ at the beginning of chromosome Pv10, identified only at the V2 stage. A second QTL, ALS 10.1^AM^, in the middle of the chromosome, showed significance at both the V3 and R8 stage. Oblessuc et al. (2012) reported the ALS10.1^UC^ QTL as responsible for the greatest effect among the seven QTL identified at the V3 stage using the UC population, and was stable for both the greenhouse and field evaluations. The ALS10.1^UC^ QTL showed an interval of 13.4 cM for the linkage map and 28.6 Mb for the physical map (Nay et al., 2019a). The BM157 marker, identified flanking ALS10.1^UC^ by Oblessuc et al. (2013), was positioned 8 cM from the ALS10.1^AM^, as well as the PVM122 marker identified at 0.6 cM from the maximum LOD mapped by Oblessuc et al. (2013) for ALS10.1^UC^. Based on this evidence, it can be concluded that ALS10.1^AM^ is the same *locus* as ALS10.1^DG,UC,GS,AS^ recognized by the BIC Genetics Committee as *Phg-5* (Nay et al., 2019a), and which was identified by López et al. (2003) in DOR364, by Oblessuc et al. (2012) in CAL143, by Keller et al. (2015) in G5686, by Bassi et al. (2017) in AND 277, by Gonçalves-Vidigal et al. (2020) in California Dark Red Kidney, and in diversity panels by Perseguini et al. (2016) and Fritsche-Neto et al. (2019). Regarding the ALS10.2^AM^ QTL, no other study reported a second QTL at the beginning of the Pv10; only Oblessuc et al. (2013) mapped ALS10.2^UC^ at the end of the chromosome. However, it should be mentioned that this is the first study involving mapping at the V2 stage, and due to the great effect of the only QTL identified, the results show that few *loci* with greater effects are associated with resistance at the beginning of plant development for the AM population.

The ALS1.1^AM^ QTL identified at the V3 stage was mapped on the same chromosome as the *Phg-1 locus* also identified in AND 277 by Gonçalves-Vidigal et al. (2011). However, the physical distance between the flanking markers in both studies was greater than 14 Mb, which can be explained by distortion of the map, due to the reduced amount of recombination of the AM population. However, the STS markers reported by Gonçalves-Vidigal et al. (2011) linked to *Phg-1* (i.e., CV542014 and TGA1.1) were not associated with ALS1.1^AM^. The third QTL, ALS 5.2^AM^, mapped for the V3 stage, was also identified in previous studies for the same stage, both in AND 277 (Bassi et al., 2017) and CAL 143 (Oblessuc et al., 2012), and in G5686 (Keller et al., 2015). The results corroborate that the resistance of CAL 143 comes from its parental line AND 277 and shows that the QTL is mainly associated with the V3 stage.

Finally, for the R8 stage evaluated under natural conditions (i.e., in the field), in addition to ALS10.1^AM^, two other QTLs were mapped. The first QTL, ALS3.1^AM^, flanked by the Marker246, was mapped at 2.22 cM distance from the FJ19 marker, which was also identified by Oblessuc et al. (2012) close to the position of the maximum LOD for the ALS3.1^UC^ QTL in the field evaluation. CAL 143 is a direct descendant of AND 277 and both significant QTLs have proximate positions under field environment conditions, which shows that, like the ALS10.1^AM^ *locus*, ALS3.1^AM^ is the same *locus* identified by Oblessuc et al. (2012) as ALS3.1^UC^ and is probably involved in defense mechanisms at advanced stages of growth plant. The second QTL, ALS11.1^AM^, was mapped on Pv11. However, only one previous study reported an ALS resistance *locus* on this chromosome (Bassi et al., 2017). The authors used the AS population and identified ALS11.1^AS^ in the opposite position to the one found in this study (Bassi et al., 2017). The development and use of SNP markers and the increased capacity of the high-throughput genotyping platform enabled the use of denser markers than had been used before. According to Li et al. (2010), simulation results indicate that the use of dense markers can improve the detection power of QTL with medium to small genetic effects. Bassi et al. (2017) used 384 SNPs, previously identified for *P. vulgaris* in selection for composition of the oligopool assay (OPA) (Müller et al., 2015). In contrast, this study used 1,091 high-quality SNPs and an additional 23 molecular markers.

### Genome-Wide Association

Linkage mapping and GWAS are both effective approaches for studying and elucidating the genetic control of quantitative traits. Linkage mapping is often used to identify the chromosomal regions controlling important agronomic traits, but due to the limited number of recombination from biparental mapping populations, few QTLs are identified and they normally have large confidence intervals (Darvasi et al., 1993; Liu et al., 2020). In contrast, GWAS rely on linkage disequilibrium (LD), which normally extends for only a short distance, and it increases polymorphism because it searches for association in a set of different background germplasms. Thus, significant QTNs tend to be found in greater number and the confidence interval is generally smaller (Kemper et al., 2012).

The results of linkage mapping using AND 277 as an Andean source of resistance for the AM population showed the complexity of the resistance mechanism during an incompatible interaction between bean and *P. griseola*. Furthermore, ALS resistance is modulated by different QTL throughout the plant growth stage, corroborating the results shown phenotypically by Pereira R. et al. (2019). The same result was observed for GWAS analysis, with significance of several QTNs for the three PGSs evaluated in the CDP of Mesoamerican origin, some QTNs being significant for more than one stage.

Among four SNPs mapped on the chromosome Pv01, the SNP ss715648599 identified at the V2 stage was 2.2 Mb distant from the first SNP belonging to the LD haplotype block in which the SNP ss715644121 significant at the V3 stage was mapped. Near this genomic region, ALS1.1^AM^ was also mapped for the V3 stage, indicating that the three *loci* identified may be the same resistance *locus* and that, in addition to being involved in resistance at both initial PGSs (V2 and V3), they were consistent with stable effects in different genetic backgrounds and environmental conditions, in both the Andean and Mesoamerican gene pools.

Two other SNPs mapped for the V3 stage on the same chromosome (ss715645868 and ss715650359) were 2.7 Mb distant from each other and were located 1 Mb from the markers CV542014 and TGA1.1, both linked to the *Phg-1 locus* (Gonçalves-Vidigal et al., 2011). More recently, Gonçalves-Vidigal et al. (2020) mapped the SNP ss715645248 flanking the *Phg-1 locus*, which was positioned between the SNPs ss715645868 and ss715650359, at an average distance of ∼1 Mb. Perseguini et al. (2016) also identified the PvM97 marker, associated with resistance to ALS in the V3 stage using a diversity panel, which was located 0.8 Mb from the SNPs ss715645868 and ss715650359. Although the *Phg-1* is recognized as a Andean source of resistance, the identification of the same region involved in the resistance of the Mesoamerican set (i.e., carioca diversity panel) is explained by the cultivar pedigree information, since Andean accessions have been widely used as sources of resistance to diseases by Brazilian bean breeders (Carvalho et al., 1998; Arruda et al., 2008; Carbonell et al., 2008). This strategy has proven to be successful in incorporating an Andean source of resistance in the Brazilian Mesoamerican elite cultivars.

On chromosome Pv02, two SNPs were mapped in different regions: ss715646000 was significant at the V3 stage, at the 48.1 Mb physical position, and ss715645817 for the R8 stage, identified at the 24.6 Mb position. Oblessuc et al. (2012) also reported the ALS2.1^UC^ QTL at the beginning of Pv02 as stable at the V3 stage and for natural conditions of field infection. In a recent study, Vidigal Filho et al. (2020) also identified a SNP (i.e., ss715648920) associated with resistance to ALS race 31-23 at 2.16 Mb from the SNP ss715646000. The SNP ss715645578 on Pv03 significant at the V3 stage is at 0.01 Mb distance from the SNP ss715645580 associated with resistance at the R8 stage. Even though both SNPs are distant from the ALS3.1^AM^ QTL, and cannot be considered as a single *locus*, the association of SNPs in LD and less than 100 Kb apart shows that the *locus* is probably involved in both intermediate and later PGSs of resistance.

Only the SNP ss715649341 was significant on Pv04, at position 43.6 Mb at the V2 stage. The *Phg-4 locus* is located in the same region, and it is also recognized as one of the largest *loci* of ALS resistance (Nay et al., 2019a). It was first mapped by Mahuku et al. (2009) linked 0 cM from marker Pv-ag004 at the V3 stage. Oblessuc et al. (2012) identified the same QTL linked to the BMd9 marker; and Keller et al. (2015), performing fine mapping of the same region, delimited the QTL flanked by the markers Marker63 and 4M439. The physical position of the markers showed that the SNP ss715649341 is positioned at 2.6, 2.5, 2.3, and 2.1 Mb for the markers Pv-ag004, BMd9, Marker63, and 4M439, respectively. Nay et al. (2019b) also identified a significant *locus* at 3.2 Mb from the SNP ss715649341 using a diversity panel composed of Andean and Mesoamerican cultivars; however, the *locus* was significant only for the *P. griseola* isolate, characterized as a 30-0 physiological race. Therefore, it was associated exclusively with an Andean source and had no potential to infect cultivars of Mesoamerican origin (Guzman et al., 1995; Pastor-Corrales and Jara, 1995). Vidigal Filho et al. (2020) also reported a significant SNP (i.e., ss715649180) associated with resistance to ALS at 0.3 Mb from the SNP ss715649341. All the other reports that mapped the *Phg-4 locus* used Andean sources of resistance (Nay et al., 2019a). Indeed, this is the first study to report the *Phg-4 locus* entirely in Mesoamerican cultivars, probably containing genomic regions introgressed from Andean genotypes used as sources of resistance.

The SNP ss715648576 mapped at position 48.5 Mb was the only significant SNP on Pv07 and was the most significant for evaluation at the V2 stage. Perseguini et al. (2016), using a Brazilian diversity panel composed of 86% Mesoamerican cultivars, also identified two markers (i.e., scaffold00111_115892 and scaffold00111) associated with ALS resistance, both at 1 Mb distance from the SNP ss715648576. Feasibly, this is one of the few resistance *loci* of Mesoamerican origin reported in the literature and may represent a source of resistance of great importance for bean breeding in countries such as Brazil, where breeding programs exploit mainly Mesoamerican germplasm (Delfini et al., 2017; Almeida et al., 2020a).

On Pv08, the SNPs ss715647412 (position 9.3 Mb) and ss715648542 (position 12.3 Mb), with a physical distance of 3 Mb from each other, were significant at the V2 stage, and because they are in LD, both can be considered as a single QTN. Perseguini et al. (2016) also identified the PvM001 marker (position 9.6 Mb) and scaffold00034_1236020 (position 15.2 Mb) in the same region, associated with ALS resistance, and three other markers at 13 Mb, associated with anthracnose resistance. Like the QTN associated with the SNP ss715648576 on Pv07, this *locus* also represents a Mesoamerican source of resistance, with great potential for use in marker-assisted selection and gene pyramiding strategies.

A third SNP (i.e., ss715647398), at the end of Pv08, was significant at the V3 stage. It was also found at 1.9 Mb from the g796 marker proposed by Miller et al. (2018) as an alternative marker to the RAPD markers mapped by Sartorato et al. (1999) for the *Phg-2 locus*. Recently, Lobaton et al. (2018) proposed the highly specific marker ALS_Chr08_CT_57798588for use in marker-assisted selection; it is 1.9 Mb from the SNP ss715647398. Nay et al. (2019b) also identified a cluster of SNPs associated with resistance to ALS at 2.6 Mb from the SNP ss715647398, and it is the only significant region for resistance both in the field and in a controlled environment. Like the previously mentioned *Phg-1* and *Phg-4*, the *Phg-2 locus* is one of the five resistance *loci* recognized by the BIC Genetics Committee and present in the Mesoamerican cultivar panel used in this study.

Finally, the last SNP, ss715646348, was mapped on Pv10, associated with resistance at the V3 stage at the 40.2 Mb position. The flanking marker of the ALS10.1^AM^ QTL was 1.3 Mb from that, showing that the *locus* identified for the CDP is the same as mapped for the AM population. Therefore, in addition to *Phg-1, Phg-2*, and *Phg-4*, the CDP has the *Phg-5 locus*, which has also been identified as a resistance *locus* of Andean origin (Nay et al., 2019b), and which is present in the set of Mesoamerican carioca cultivars.

### Genes Involved in ALS Resistance

According to Schmutz et al. (2014), recombination events in centromeric and pericentromeric regions of the bean genome are limited, leading to the formation of large gene blocks in LD, which results in the identification of QTLs with a large confidence interval for these regions in segregating populations (Blair et al., 2018). This explains the greater interval of the ALS1.1^AM^ and ALS3.1^AM^ QTL, both positioned at the pericentromeric region. The same was observed for the QTN mapped in these regions; however, the QTL interval is still significantly larger than the QTN. The smaller QTN interval is mainly due to the large number of recombinations that accumulate over the generations of each cultivar that composed the CDP, allowing identification of candidate genes in a more precise way, mainly in the pericentromeric and centromeric regions.

Among the candidate genes involved in plant disease resistance, the R genes are the most important type of putative resistance-associated genes (Wu et al., 2017). Most of the R genes encode intracellular proteins that contain a nucleotide-binding (NB) site and a C-terminal leucine-rich repeat (LRR) domain (Meziadi et al., 2016). NB-LRR (NL) can be split into two subclasses (Ameline-Torregrosa et al., 2008): the N-terminal domain with Toll/Interleukin-1 Receptor homology (TIR-NB-LRR, or TNL), and the N-terminal coiled-coil motif (CC-NB-LRR, or CNL). One of the largest and most variable multigene families in plants is the one that encodes NB-LRR proteins, which frequently compose complex clusters with tightly linked genes (Meyers et al., 2003; Jupe et al., 2012). In beans, most of the R gene clusters are at the end of the chromosomes and have larger size than in other species (Meziadi et al., 2016).

Schmutz et al. (2014) identified 376 NL genes, of which 106 were classified as TNLs and 108 as CNLs. The authors reported the presence of some clusters containing more than 40 NL genes in the bean genome. Within the QTN2.1 interval, a large cluster containing 21 NL genes was identified, and another cluster with 5 NL genes was positioned within the QTN10.1 interval. Close to QTN4.1, another set with 15 NL genes was identified; and QTN8.3 (*Phg-2*), mapped at the end of the Pv08, had a close cluster with 15 NL genes (Figure 4). In a recent study, Gil et al. (2019) carried out fine mapping for this *locus* and reported the same cluster (NL) linked to the *Phg-2 locus* as the best putative candidate genes for ALS resistance. Nay et al. (2019b), in a study involving different haplotypes of the *Phg-2*, also reported NL genes as the most likely candidates.

Although the results corroborate previous studies regarding the importance of R genes in control of ALS resistance, the presence of these genes close to 13 of the 18 *loci* mapped shows that the studies reporting a single *locus* involved in the resistance mechanism to ALS may be a consequence of low statistical power in identifying *loci* of lesser effects. Oblessuc et al. (2015), when selecting putative genes resulting from the fine mapping performed by Oblessuc et al. (2013) for transcriptional modulation analyses, also reported many NL genes in the QTL interval ALS10.1^UC^ and showed that some R genes are consistently up-regulated in the susceptible cultivar, suggesting that the R gene may be involved in susceptibility to ALS, making the host-pathogen interaction even more complex.

Considering the PGSs evaluated, different resistance *loci* and degrees of susceptibility were observed for both sets. Interestingly, no gene with a genetic annotation causally linked to disease resistance was allocated in the ALS10.2^AM^ QTL interval mapped for the V2 stage, in which the AM population had the minimum degree of resistance. However, 13 resistance genes were identified for the ALS10.1^AM^ mapped for the V3 and R8 stage. Moreover, four resistance genes were identified for the ALS1.1^AM^ mapped for the V3 stage and another 17 for ALS5.1^AM^ (Supplementary Table 3). For CDP, the highest number of putative resistance genes was also identified at the R8 stage (Table 5), a result that can be explained by the “priming” effect, in which plants that are naturally exposed to other biotic and abiotic stresses during a field experiment can induce alertness, activating the basal defense signaling pathways controlled by a genetic induction chain not limited only to genes with greater effects (Borges et al., 2017).

Future studies involving differential expression with the candidate genes reported in Supplementary Table 3 should be conducted to identify the genes with the greatest effect on ALS genetic resistance, with emphasis on Phvul.003G269200 (TIR-NBS-LRR class), which was identified in the interval of QTN3.2 (V3) and in QTN3.1 (R8). In addition, the markers associated with the resistance *loci* represent a powerful tool for crop breeding, which can be applied in studies on germplasm bank screening, selection of parents holding resistance alleles, and marker-assisted and backcross selection (Rocha et al., 2012; Rezene et al., 2019; Almeida et al., 2020b).

## Conclusion

This is the first mapping study to identify *loci* associated with ALS resistance at different plant growth stages of diverse genotypes from different genetic backgrounds using two mapping approaches. The quantitative inheritance of the disease was confirmed by the identification of several *loci* associated with different phenological stages and different environmental conditions. The genetic basis of ALS resistance is modulated by several genomic regions throughout the plant growth stages; however, some *loci* are stable for both early and late stages. Genomic regions identified for more than one PGS, such as QTN3.1 - QTN3.2, or associated with different population, such as ALS10.1^AM^ and QTN10.1, are those with the greatest potential for crop breeding, enabling the development and use of molecular markers to select advanced lines for durable ALS resistance. In addition to *Phg-1*, AND 277 has the *Phg-5 locus*, the first time this has been reported in the CAL143 derived cultivar. Several of the *loci* identified validated regions of previous studies, and four of the five *loci* of greatest effects recognized by the BIC Genetics Committee were identified in the carioca diversity panel (*Phg-1*, *Phg-2*, *Phg4*, and *Phg-5*).

## Data Availability Statement

The original contributions presented in the study are included in the article/Supplementary Material, further inquiries can be directed to the corresponding author/s.

## Author Contributions

LLB-R, AC, JP, and CA designed the study, and LLB-R was responsible for project funding. CA and JC conceived the structure of the manuscript, and CA wrote the initial manuscript. CA, JC, GB, IS, JG, and FP conducted the experiments, and QS conducted molecular genotyping. CA, GS, and CT performed the data analyses. FP supported phenotyping curation and AG, the accuracy analyses. LLB-R, AC, and SC supported data curation and reviewed the manuscript. All the authors have revised and approved the final version of the manuscript.

## Conflict of Interest

The authors declare that the research was conducted in the absence of any commercial or financial relationships that could be construed as a potential conflict of interest. The handling editor declared a past co-authorship with several of the authors CA, JC, SC, AC, QS, LLB-R.

## Footnotes

1 http://sourceforge.net/projects/bbmap/

2 http://www.ncbi.nlm.nih.gov/sra

## Supplementary Material

Supplementary Figure 1 Linkage map estimated by 1,091 SNPs obtained by GBS and another 23 molecular markers using the AM population composed of 91 BC_2_F_3_ lines originating from the inter-gene pool cross (AND 277 × IAC-Milênio).

Supplementary Table 1 Details of the 125 carioca cultivars that make up the CDP: name, grain size (mm), commercial classification, institution of origin, adjusted mean (BLUE), normalized data of evaluation of resistance to angular leaf spot at different plant growth stages (V2, V3, and R8 stages), genealogy, and genotypic matrix.

Supplementary Table 2 Phenotypic and genotypic matrices of the AM segregating population composed of 91 BC_2_F_3_ lines: marker name, position (cM and pb), and alleles for 1,091 SNPs and another 23 molecular markers used in the AM linkage map.

Supplementary Table 3 Genetic annotation of each *locus* mapped for the three evaluations of resistance to angular leaf spot (V2, V3, and R8) through both approaches: GWAS and linkage mapping. First table is composed only of genes with genetic annotations related to disease resistance identified in the confidence interval of each marker. All genes present in the confidence interval of each *locus* are present in separate tabs.
