# Supplementary figures and images for: Angular Leaf Spot Resistance Loci Associated With Different Plant Growth Stages in Common Bean
Source: Front Plant Sci. 2021 Apr 13;12:647043. doi: 10.3389/fpls.2021.647043 (PMC8078856; doi:10.3389/fpls.2021.647043)

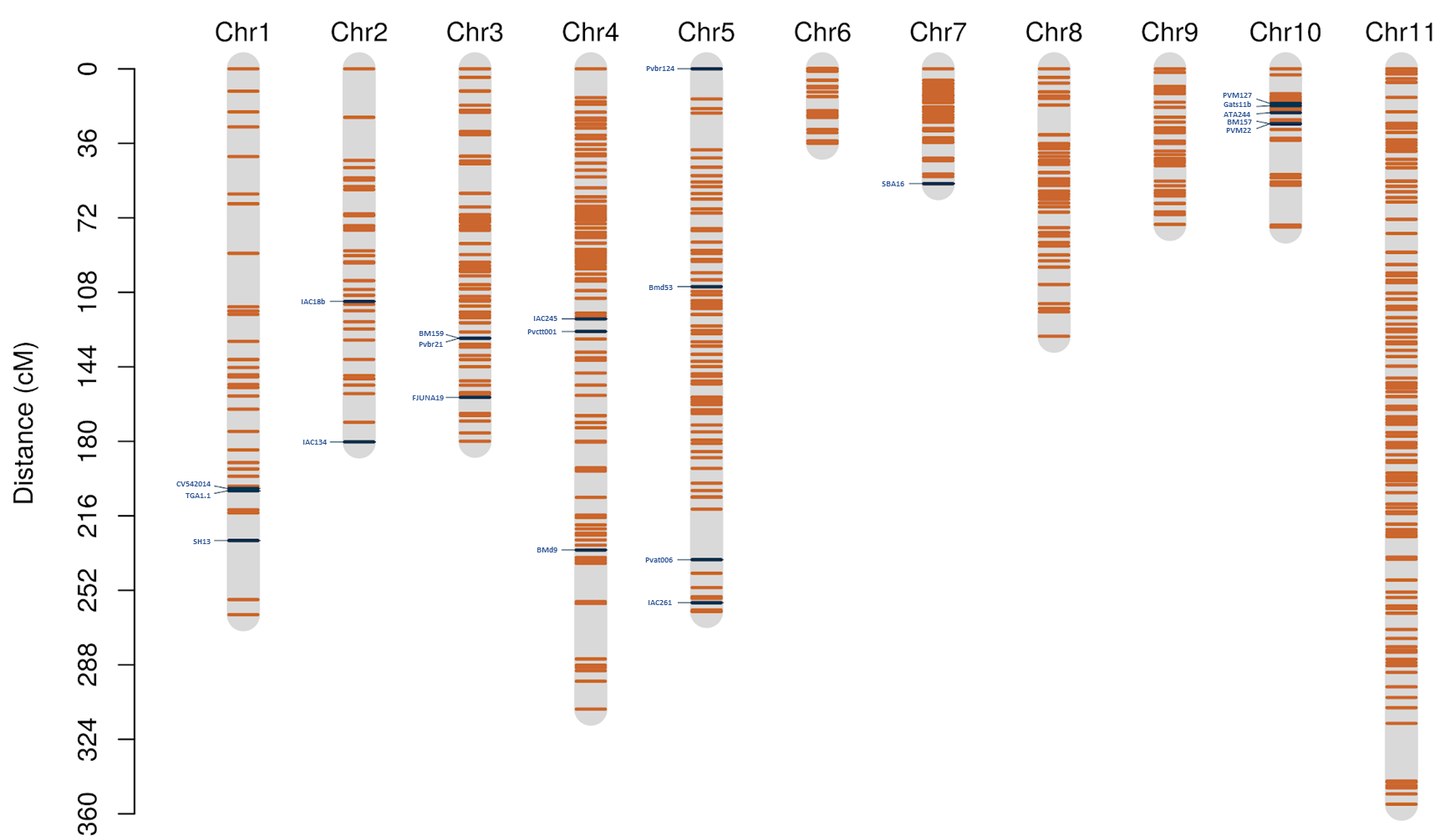

Supplement: Supplementary Figure 1 — Linkage map estimated by 1,091 SNPs obtained by GBS and another 23 molecular markers using the AM population composed of 91 BC2F3 lines originating from the inter-gene pool cross (AND 277 × IAC-Milênio). [file Image_1.TIF]
